# Down-staging of obesity one year after bariatric surgery: a new proposal of Edmonton obesity staging system

**DOI:** 10.3389/fendo.2023.1147171

**Published:** 2023-07-21

**Authors:** Giulia Quinto, Silvia Bettini, Daniel Neunhaeuserer, Francesca Battista, Gabriella Milan, Andrea Gasperetti, Marco Vecchiato, Roberto Vettor, Andrea Ermolao, Luca Busetto

**Affiliations:** ^1^ Sports and Exercise Medicine Division, Regional Center for the Therapeutic Prescription of Exercise in Chronic Disease, Department of Medicine, University of Padova, Veneto Region, Italy; ^2^ Department of Medicine, Center for the Study and Integrated Treatment of Obesity (CeSTIO), Internal Medicine 3, University of Padova, Veneto Region, Italy

**Keywords:** cardiorespiratory fitness, cardiopulmonary exercise testing, EOSS, obesity therapy, obesity classification

## Abstract

**Background:**

Different approaches are used to classify obesity severity. The Edmonton Obesity Staging System (EOSS) considers medical, physical and psychological parameters. A new modified EOSS with a different functional evaluation method, measuring Cardiorespiratory Fitness (CRF), has been recently proposed, EOSS-CRF. Bariatric surgery (BS) is one of the most efficient treatments of obesity and all aspect of related disorders. No studies have yet applied EOSS-CRF after BS. Therefore, the aim of this study was to evaluate modifications in EOSS and EOSS-CRF before and after BS.

**Methods:**

This observational study finally enrolled 72 patients affected by obesity. A multi-disciplinary assessment in order to evaluate eligibility to surgical treatment has been performed, including anamnesis, physical evaluation, anthropometric data measurement, biochemical blood exams and cardiopulmonary exercise testing. One year after BS the same protocol was applied. Patients have been classified according to EOSS and EOSS-CRF before and one year after BS.

**Results:**

After BS, patients categorized in classes associated to severe obesity (EOSS ≥ 2 or EOSS-CRF ≥ 2) reduced significantly. Using EOSS, patients without functional impairment were 61% before surgery and 69% after BS (p=0.383). Using EOSS-CRF, patients considered without functional impairment were only 9.7% before BS; this percentage significantly raised to 50% after BS (p<0.001). The impact of functional domains before and after BS is different in grading patients in EOSS and EOSS-CRF, respectively.

**Conclusions:**

Improvements obtained after BS are adequately summarized by EOSS and EOSS-CRF. The EOSS-CRF grading method for functional impairment seems to better reflect the known amelioration obtained after BS. Objective measurements of CRF may provide additional value to classify severity of obesity, also in the follow-up after BS.

## 1 Introduction

Obesity is widely recognised as a chronic disease and in the last decade it has spread to more than 2 billion adults, leading to a reduced life expectancy worldwide (1, 2). Indeed, obesity is considered a life-threatening condition and a major risk factor for non-communicable diseases (3). It is associated with an increased risk of dyslipidaemia, arterial hypertension, and type 2 diabetes mellitus (T2DM), as well as with higher rates of cardiovascular and metabolic mortality (4, 5). Osteoarthritis is another known complication of this chronic disease (6). Moreover, patients affected by obesity often have a considerable functional impairment which could significantly alter their quality of life (7, 8).

To classify obesity and assess the related cardiovascular risk, Body Mass Index (BMI) and waist circumference (WC) are preferably used in clinical practice as surrogate measures of body and visceral fat, respectively (9). Although both methods are easily reproducible, they are limited related to the inability to assess the presence of comorbidities, functional capacity and quality of life as well as other prognostic contextual factors that may characterize clinical risk and influence patients’ management (10). For these reasons, the Edmonton Obesity Staging System (EOSS) provides a five-stage system for obesity classification, considering medical, physical and psychological parameters, allowing clinicians to monitor comorbidities associated with excess weight (11). Moreover, EOSS is able to identify subjects at high risk of mortality (12, 13). However, it provides a wide flexibility in the assessments of physical disability and psychological status, where clinicians can give a subjective level of disease severity. Therefore, in order to overcome this limitation, our group recently proposed the EOSS-CRF, in which patients’ functional capacity is objectively assessed through Cardiopulmonary Exercise Testing (CPET), by measuring Cardiorespiratory Fitness (CRF) expressed as peak oxygen consumption (VO_2_peak [mL/Kg min]) (14). EOSS-CRF grades functional impairment in mildly, moderately or severely compromised, which may influence clinical decision making (14).

The outcome of obesity management is usually reported as degree of weight loss, improvement/resolution of obesity-related diseases, improvement of physical function and quality of life, each of them considered separately. In the context of obesity as a chronic disease affecting multiple clinical domains, it would be useful to physicians and patients to know if the prescribed treatment is able to reduce the global burden of the disease; in other words, to know if the treatment leads to a down-staging of obesity severity. Bariatric surgery (BS) is currently one of the most efficient treatments to obtain and maintain weight loss in the long-term (15, 16). Also, after BS, improvement and/or remission of the main complications related to weight gain, such as T2DM, arterial hypertension, obstructive sleep apnoea syndrome (OSA) and non-alcoholic fatty liver disease (NAFLD) have been described (17). Moreover, BS is considered the most efficient intervention for severe obesity also regarding physical functioning and performance (18, 19). Although patients’ absolute muscle strength, usually measured by handgrip test, has been described as reduced or unchanged after BS, relative muscle strength (expressed as the ratio between the handgrip strength and BMI), has been shown to improve between 6 and 12 months post-surgery, probably due to a progressive decrease in fat infiltration of skeletal muscles after the initial lean mass loss (7, 20). Furthermore, after BS, improvement in abilities to perform activities of daily living was described (21). Considering CRF, different studies reported a significant increase in VO_2_peak relative to body weight and in cardiorespiratory efficiency (18, 22–24). Evaluating the decreased or unchanged absolute values of VO_2_peak after BS, data may suggest that the improvement in CRF is mainly due to weight loss and changes in body composition (18). However, also impaired peripheral oxidative muscle metabolism has been discussed after BS (7). More recently, evaluating short-term versus long term data, Neunhaeuserer et al. showed that 18 months after BS an improvement in overall aerobic capacity could be achieved (22).

Therefore, BS improves the overall health status and the application of comprehensive staging systems, such as EOSS and EOSS-CRF, would be important to better show efficacy of obesity treatments. Thus, the aim of this study is to evaluate the impact of BS on the EOSS and EOSS-CRF classification in patient affected by obesity, focusing on the importance to objectively defined not only medical but also functional impairment after surgically-induced weight loss.

## 2 Materials and methods

### 2.1 Population and study design

In this observational study, patients with obesity were consecutively assessed at the Centre for the Study and Integrated Treatment of Obesity, University Hospital of Padua, Italy, in the period between 2014–2020. Ninety-eight patients were evaluated. All patients underwent a multi-disciplinary evaluation according to a standardized clinical protocol, in order to examine eligibility to surgical treatment for obesity (T_0_ evaluation). This clinical pathway includes an overall assessment with anamnesis, physical evaluation, anthropometric data measurement, biochemical blood analyses and CPET; the latter has been performed by Sports and Exercise Medicine Specialists at the Sports and Exercise Medicine Division of the University of Padua. Subsequently, patients underwent BS, using the most appropriate surgical technique according to the specific case. One year after BS, patients were re-evaluated with the same protocol (T_1_ evaluation). Only patients who presented all data to stage according to EOSS and EOSS-CRF classes before and after BS were included. Further exclusion criteria were previous BS, major contraindication to CPET, potentially end-stage functional limitation unable to perform CPET, T_1_ evaluation more than 16 months after surgery. Finally, 72 patients were included.

All subjects gave written informed consent in accordance with the Declaration of Helsinki. The protocol was approved by the “Padua Ethical Committee for Clinical Research” (2892P, 10/06/2013).

### 2.2 Anthropometric and Biochemical assessment

Height was measured to the nearest 0.005 m using a stadiometer. Body weight was determined to the nearest 0.1 kg using a calibrated balance beam scale. BMI was calculated as weight (kg) divided by height squared (m^2^). Anthropometric data were taken with subjects wearing only light clothes without shoes. Absolute weight loss (WL) was expressed as [weight pre-BS (kg)] – [weight post-BS (kg)]. Relative WL was expressed as [absolute WL (kg)]/weight pre-BS (kg)] *100.

All blood tests were performed after 8-h fasting. For each patient we collected full blood count, fasting plasma glucose (FPG), lipid profile [total cholesterol (TC), High Density Lipoprotein-cholesterol (HDL), triglycerides (TG)], alanine aminotransferase (ALT), aspartate aminotransferase (AST) and gamma glutamiltrasferase (GGT). Low Density Lipoprotein-cholesterol (LDL) was calculated according to Friedewald (25). At T_0_, in patients without known diabetes, a 3-h oral glucose tolerance test (OGTT) was performed monitoring blood glucose and insulin plasma levels after glucose load (75 g) (26).

### 2.3 Cardiopulmonary exercise testing

As we have extensively described in our previous study (14), incremental and maximal CPET was performed in both evaluations preferentially on treadmill (COSMOS, T170 DE-med model) with the modified Bruce protocol; bicycle ergometer with individually adapted protocols was used in patients with orthopaedic limitations or gait disturbances. ECG, arterial blood pressure, and peripheral oxygen saturation were continuously monitored at rest, during exercise as well as in the recovery phase. Ventilatory and gas exchange measurements were sampled breath-by-breath and measured by a low- resistance turbine and mass spectrometry, respectively (Masterscreen CPX Jaeger, Carefusion, Hoechberg, GE system) (27). Criteria of exhaustion were a Borg rating of perceived exertion ≥ of 18/20, associated with either a maximal heart rate (HR) ≥ 85% of predicted (220 bpm – age) or a peak Respiratory Exchange Ratio (RER) > 1.10 (28, 29). Patients were verbally encouraged to reach maximal exertion. Main parameters of CRF and efficiency were obtained, also including VO_2_peak, VO_2_peak/Kg and the Oxygen Uptake Efficiency Slope (OUES) (30).

### 2.4 EOSS and EOSS-CRF classifications

Obesity-related comorbidities and/or complications were evaluated in order to classify patients in different EOSS and EOSS-CRF classes by medical domains. Patients’ glycaemic profile was divided in normal glycaemia, pre-diabetes (pre-DM) (impaired fasting glycaemia and/or impaired glucose tolerance at the OGTT) and T2DM (26). Diagnosis of arterial hypertension, dyslipidaemia and OSA was based on recent guidelines (31–33). Psychiatric symptoms were also collected. For the EOSS classification, functional impairment was classified using not quantitative parameters, but considering the presence of clinically relevant osteoarthritis, limitations in activities of daily living and/or impairment of well-being (i.e. being able to tie shoes or to do housework), evaluated through anamnesis. Moreover, patients who were not able to perform a standard treadmill CPET because of their functional limitations and needed a bicycle ergometer test, were classified as moderately limited. Patients with end-stage organ damage at one or more joints, that needed previous surgery, were considered affected by severe functional limitation (14). As previously described in detail, for the EOSS-CRF classification, functional capacity expressed as VO_2_peak/Kg and relative percentiles of the FRIEND registry have been used to grade the functional limitation in mild, moderate or severe impairment (14, 34). We categorized patients to EOSS and EOSS-CRF classes respectively at T_0_ and T_1_ by using the highest-stage risk factor in each domain for each patient.

### 2.5 Statistical analysis

Data have been analysed with IBM SPSS Statistics (IBM Corp. Released 2017. IBM SPSS Statistics for Windows, Version 25.0. Armonk, NY: IBM Corp.). All continuous variables were analysed for normality by the Shapiro-Wilk test and, based on their distribution, T_0_-T_1_ comparisons were performed by paired samples Student’s t test or Mann-Whitney test, respectively. Variables expressed as percentage were compared with chi-square test or exact Fisher’s test depending on their numerousness. P value ≤ 0.05 was considered statistically significant.

## 3 Results

### 3.1 Baseline characteristics

Seventy-two patients affected by obesity who underwent BS were enrolled (BMI 42.21 ± 6.25 Kg/m^2^, range 34.93 – 64.32 kg/m^2^). T_1_ evaluations were performed 13.11 ± 2.85 months after BS. Sixty-nine patients underwent laparoscopic sleeve gastrectomy (95.8%), while three patients underwent roux en y gastric bypass (4.2%). Anthropometric characteristics and the prevalence of comorbidities before and after BS are shown in **Table 1**. Absolute WL was 38.55 ± 14.77 kg and relative WL was 31.89 ± 8.39%.

**Table 1:** Anthropometric data, clinical outcomes and cardiopulmonary evaluation in 72 patients with obesity.

	*Pre-BS*	*Post-BS*	*p*
*Sex (M/F)*	28/44	28/44	*-*
*Age (years)*	47.36 ± 9.95	48.74 ± 9.87	*-*
*Height (cm)*	166.73 ± 10.34		*-*
*Weight (kg)*	123.65 ± 25.51	85.10 ± 18.71	<0.001
*BMI (kg/m^2^)*	44.21 ± 6.55	30.40 ± 4.85	<0.001
***Comorbidities***			
*T2DM [N(%)]*	25 (34.7)	9 (12.5)	<0.001
*Pre-DM [N(%)]*	27 (37.5)	7 (9.7)	0.001
*HYPT [N(%)]*	44 (61.1)	24 (33.3)	<0.001
*DLP [N(%)]*	48 (66.7)	44 (61.1)	0,488
*OSA [N(%)]*	25 (34.7)	9 (12.5)	<0.001
***CPET parameters***			
*VO_2_ peak/Kg* *(mL/Kg min)*	19.39 ± 4.11	24.23 ± 5.82	<0.001
*VO_2_ peak (L/min)*	2.395 ± 0.670	2.073 ± 0.605	<0.001
*OUES (mL/logL)*	2519.9 ± 683.6	2017.3 ± 578.8	<0.001
*RER peak*	1.16 ± 0.10	1.26 ± 0.12	<0.001
*HR max (bpm)*	148.81 ± 19.94	152.83 ± 20.66	0.112
*HR peak predicted per age (%)*	86.22 ± 10.01	87.46 ± 16.64	0.608

Continues variables are expressed as mean ± SD. Differences between before and one-year after-BS are expressed as absolute value. M, male; F, female; BS, bariatric surgery; BMI, body mass index; T2DM, type-2 diabetes mellitus; HYPT, arterial hypertension; DLP, dyslipidaemia; OSA, obstructive sleep apnoea syndrome; CPET, cardiopulmonary exercise test; VO_2_peak/kg, Weight-adjusted peak of oxygen consumption; VO_2_peak, peak of oxygen consumption; OUES, oxygen uptake efficiency slope; RER, respiratory exchange ratio; HR, heart rate; ns, not significant; -, not applicable. P value ≤ 0.05 was considered statistically significant.

After BS, nine (12.5%) patients reached a BMI<25 kg/m^2^, while 28 (38.9%) patients improved their status to overweight with a BMI from 25.00 to 29.99 kg/m^2^. Thirty-five (48.6%) patients remain affected by obesity with a BMI≥30 kg/m^2^. Most obesity-related comorbidities and/or complications significantly reduced after surgery, except for dyslipidaemia. Comorbidities remissions after BS in patients who reach different BMI classes were described in **Table 2**. No significant differences were observed in the rates of improvements of comorbidities according to the reached level of BMI.

**Table 2:** Comorbidities remissions after bariatric surgery (BS) in patients who reached different BMI classes.

	Normal weight after BS	Overweight after BS	Obesity after BS	p-for-trend*
T2DM (%)	66.7%	45.5%	81.8%	0.205
Pre-DM (%)	100%	90.0%	93.3%	0.874
HYPT (%)	60.0%	47.1%	50.0%	0.771
DLP (%)	33.3%	31.3%	26.1%	0.898
OSA (%)	66.7%	75.0%	54.5%	0.653

Data are expressed as percentage of patients going into remission of a specific comorbidity. BMI, body mass index; B T2DM, type-2 diabetes mellitus; HYPT, arterial hypertension; DLP, dyslipidaemia; OSA, obstructive sleep apnoea syndrome. *comparison between all groups.

The CPET evaluation showed a significant improvement in functional capacity expressed as VO_2_peak/kg, with an average difference of 4.84 ± 3.89 mL/kg min. Absolute VO_2_peak and OUES significantly decreased.

### 3.2 EOSS and EOSS-CRF classifications

**Figure 1:**
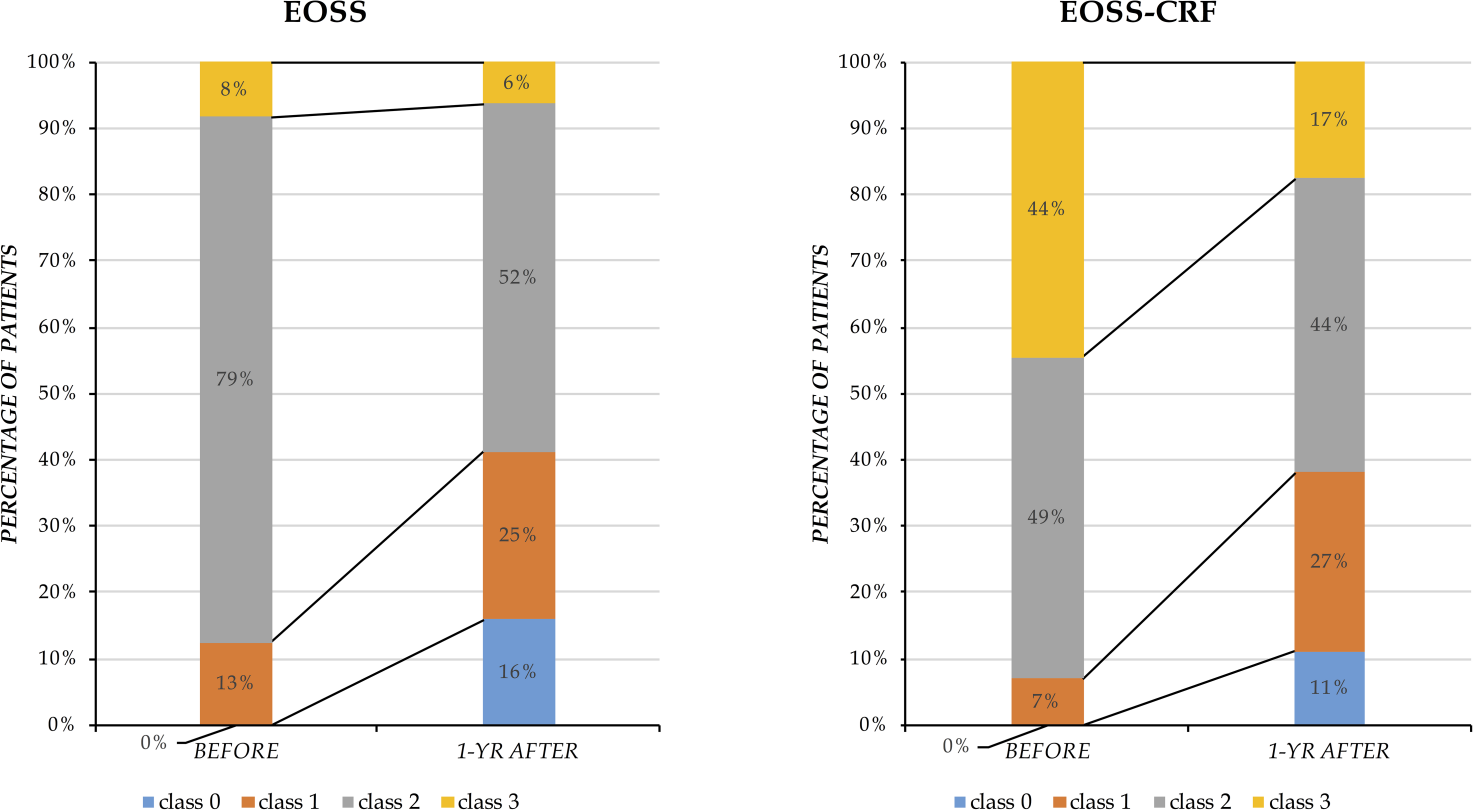
Distribution of patients classified by EOSS and EOSS-CRF before and after bariatric surgery. Results are presented as percentage of patients distributed in different classes (y-axis) before and one year after bariatric surgery (x-axis).

EOSS and EOSS-CRF were used to classify patients before and after BS (**Figure 1**). Patients categorized in classes EOSS≥ 2 or EOSS-CRF≥ 2 were found significantly reduced in numbers after BS. Particularly, using EOSS, the percentage of these patients decreased from 88% to 56%, while using EOSS-CRF, from 93% to 58%. Furthermore, distributions in different classes behaved differently according to the two classifications: in EOSS, patients in class 2 significantly changed, reducing from 79% to 50%, while patients in class 3 remained substantially unchanged (8% versus 6%). On the other hand, in EOSS-CRF, patients in class 2 did not change significantly (49% versus 43%), and the most difference can be observed in class 3, where patients diminished from 44% to 15%.

### 3.3 Functional impairment

**Figure 2:**
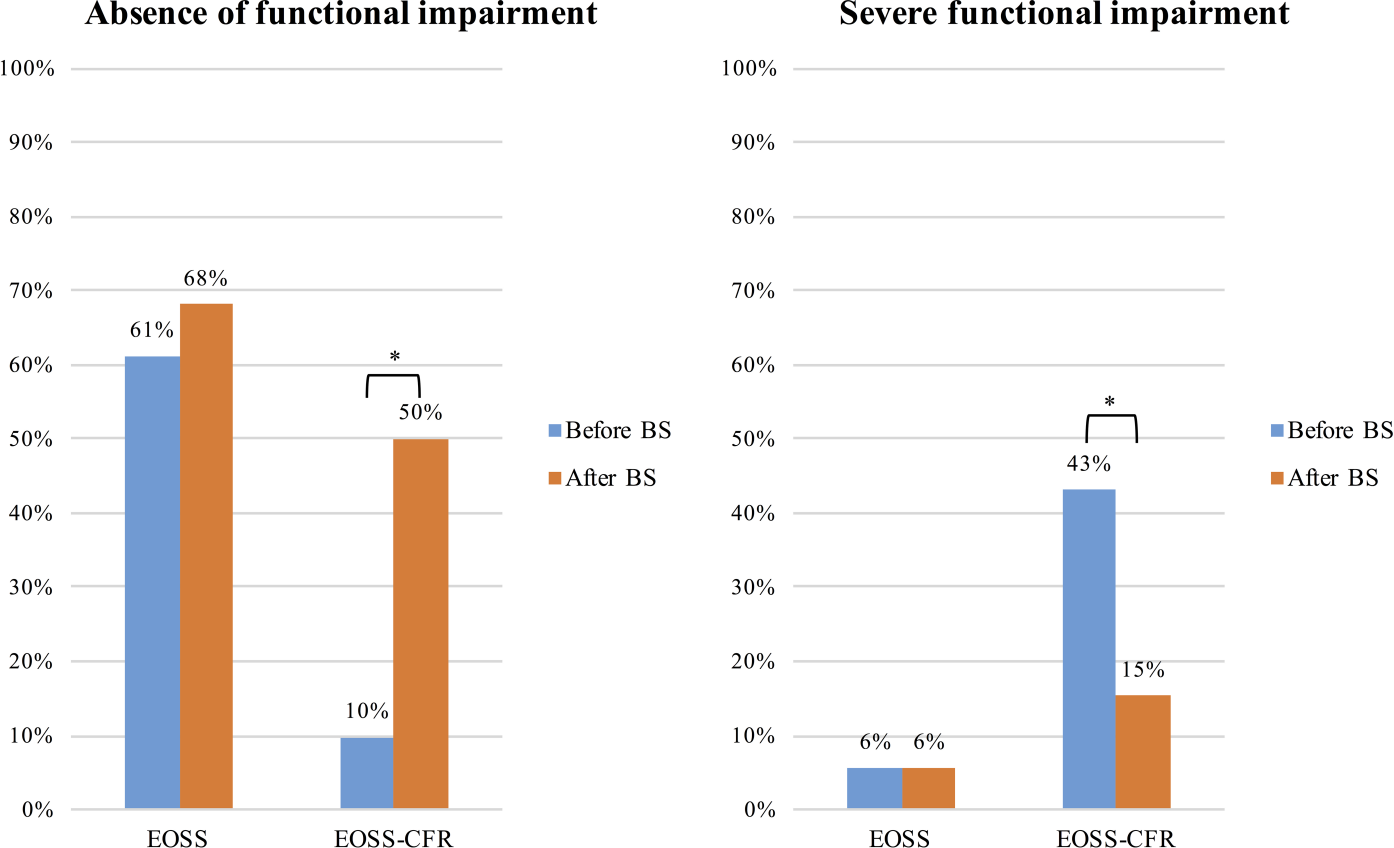
Presence of functional impairment before and after bariatric surgery according to the two different classifications, EOSS and EOSS-CRF. Results are presented as frequency (percentage) of patients without any, and those with severe functional impairment before and after bariatric surgery. *p<0.05.

The presence of functional impairment according to the different classifications before and after BS is shown in **Figure 2**. Using EOSS, more than half of the patients were considered as without functional impairment before BS and there was not difference compared with patients after BS (p=0.383). According to EOSS-CRF, patients without a functional impairment, graded using VO_2_peak/Kg were only 9.7%. This percentage significantly raised to 50% (p<0.001) after BS. Moreover, the prevalence of patients with a moderate or severe functional impairment classified through EOSS-CRF significantly reduced after surgical weight loss (from 18% to 6% with moderate functional impairment, p=0.02; from 43% to 15% with severe functional impairment, p<0.001).

### 3.4 Role of different domains in patient classifications after BS

**Figure 3:**
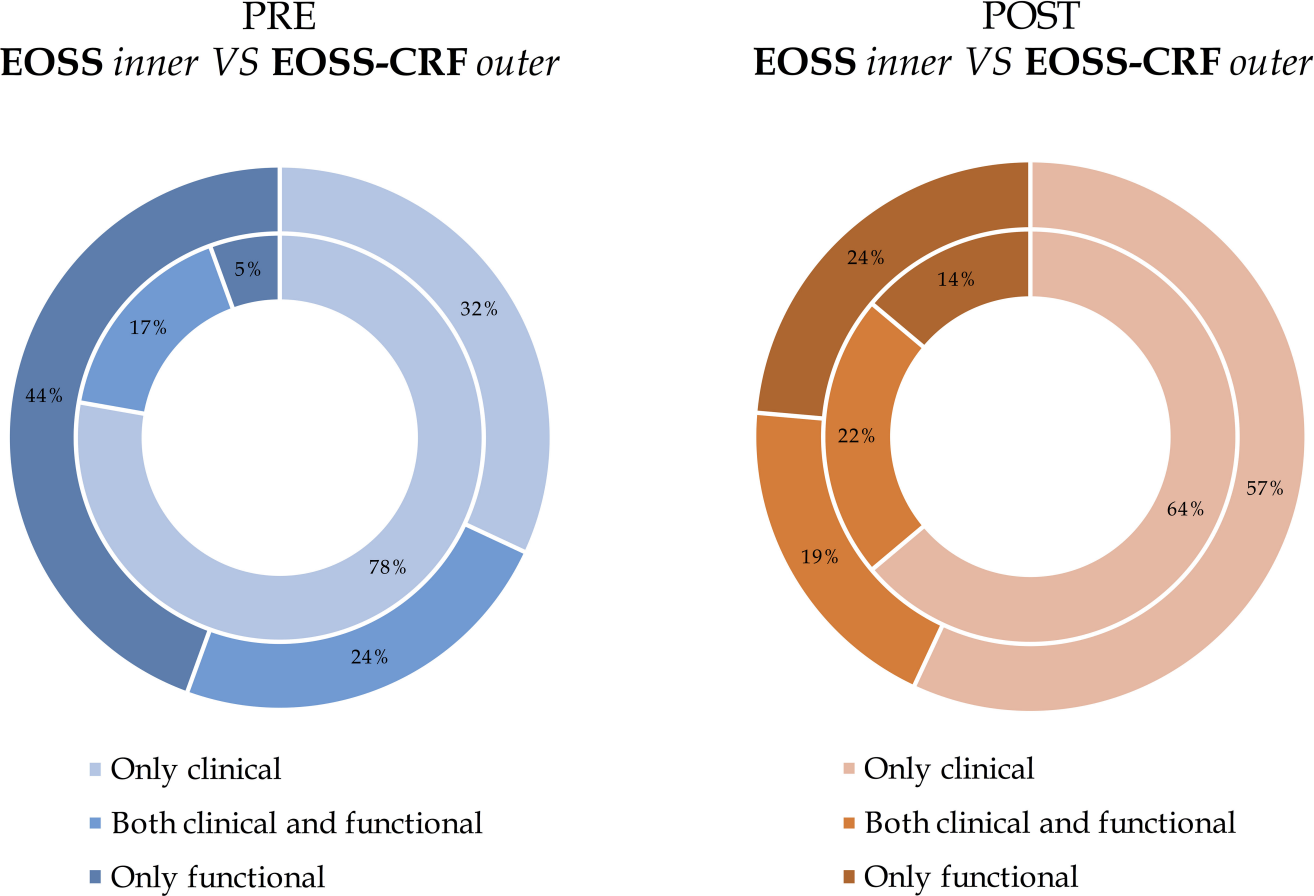
The impact of different domains on EOSS and EOSS-CRF before and after bariatric surgery. The reasons were only clinical, both clinical and functional or only functional. Results are presented separately for evaluations before and after bariatric surgery and they are expressed as frequency (percentage) comparing EOSS (inner circle) and EOSS- CRF (exterior circle).

To better understand the role of clinical and functional domains and thus the impact of the different approaches, **Figure 3** shows the reasons why patients were assigned to classes, visually comparing EOSS and EOSS-CRF, before and after BS. In other words, clinical and functional determinants were analysed for the assignment of patients to EOSS and EOSS-CRF classes, respectively. The impact of the functional domain before and after BS was different in EOSS and EOSS-CRF. Moreover, in the classical EOSS, the functional domain distinguishes 4% of patients before BS and 16% after BS. CRF classifies alone 44% of patients before BS and 27% after BS for the new EOSS-CRF. Focusing on the most severe included class, the functional domain in EOSS classifies 60% of class 3 patients, becoming 100% after BS. On the other hand, EOSS-CRF classifies 94% of class 3 patients before BS, remaining 91% after BS.

## 4 Discussion

In our study, we confirmed the short-term efficacy of BS in the management of severe obesity (15, 16). We observed an average relative WL of 31.89 ± 8.39%, in line with previous studies that reported a WL one year after BS of about 30-35% (35, 36). Thus, using only BMI to classify this chronic disease, BS is particularly effective, especially when compared with medical therapy alone (16). Moreover, BS is effective in reducing both comorbidities and/or complications and patient’s ability to perform activities of daily living (21, 37, 38). However, the improvement in functional capacity expressed as VO_2_peak/kg after BS is more debated (18). Our data support the previous findings described in literature: a significant improvement of VO_2_peak/kg, associated with a reduction in absolute VO_2_peak and cardiorespiratory efficiency. The reduction in these absolute values is clinically related to the loss of muscle mass after BS, while the substantial increase in VO_2_peak/kg, which is maintained for at least 18 months, is a marker of improved functional physical impairment (22, 39). Furthermore, CRF expressed as VO_2_peak/kg is a strong independent predictor of cardiovascular risk and all-cause mortality (40–42).

When combining all the evaluations performed, our results show that the improvements obtained after BS are also highlighted and adequately summarized by the latest classifications of obesity, i.e. EOSS and EOSS-CRF. These classifications had been designed to provide a holistic and more comprehensive assessment of all different comorbidities and/or obesity-related complications, but no study has yet compared these classifications before and after BS (11, 14). In our study, the number of patients still affected by severe obesity (EOSS class ≥2 or EOSS-CRF class ≥ 2) (43), was significantly reduced after BS; by 30% if assessed by EOSS was applied, and by 32% when EOSS-CRF was applied. Indeed, these scores show how complications and functional impairments related to weight excess, can be improved with BS (44). In the context of obesity as a chronic condition, staging systems have been developed to monitor the medical and functional status during the course of disease (11, 14). In our study, the application of the EOSS and EOSS-CRF staging systems after substantial weight loss seems to demonstrate that effective obesity management is able not only to halt the progression of the disease, but also to revert it to less advanced stages (down-staging).

Since the methodology between both grading systems is different (11, 14), the two classifications result in different categories. In particular, the evaluation of functional impairment through anamnestic data collection of orthopaedic limitations, difficulties in activities of daily living and well-being is not standardised and difficult to apply. As a possible consequence, functional impairment in the EOSS classification seems not to be significantly addressed before and after BS. Moreover, the subgroup of patients affected by severe functional impairment remained unchanged after BS, because of anamnestic data of previous joint surgery, expression of end-stage organ damage, did not change after BS. On the other hand, functional capacity, objectively measured through CRF and then categorised in mild/moderate/severe impairment, evaluates in a single parameter all aspects of functional limitation during daily living, overcoming subjectively assessed anamnestic data (45). Moreover, this grading method seems to better reflect the known amelioration that BS has demonstrated in all aspect of performance and health indices (18). This new approach for categorizing disability, using the FRIEND registry percentiles as normal values to categorise patients’ CRF, has recently been successfully applied to other disorders (46).

Focusing on the functional impairment of EOSS-CRF, the number of patients affected by moderate or severe functional disabilities significantly reduced after BS and patients with no functionally significant alteration of CRF considerably raised from 9.7% to 50%. Conversely, patients affected by mild functional impairment did not change. Thus, BS is likely to improve functional impairment, particularly in more advanced states of disability, leading to patients having mild or no physical dysfunctions. However, it is well known that, also due to the lean mass loss observed especially during the first year after BS, it is important to combine surgical treatment with exercise therapy to maintain and improve CRF and the overall health status (47).

Before BS, the functional domain of the EOSS classification determines only 4% of patients’ distribution, regardless of classes. Using EOSS-CRF, the impact raises to 44%. Previously, it has already been described how objectively measured CRF, as marker of physical impairment, plays a major role in determining clinically and prognostically relevant disease severity in obesity. Bettini et al. showed how this new categorisation method changes particularly the classification of the most severe stages of obesity. The impact of only clinical parameters to stage for obesity severity has been markedly decreased by applying the EOSS-CRF, particularly for class 3 (88.2% versus 6.8%). Indeed, patients were predominantly assigned to EOSS-CRF class 3 for severe functional impairment (85.5%), while the previous functional markers determined EOSS class 3 in only 11.8% (14).

After BS, the overall importance of the functional domain in determining classifications seems to be similar than before surgery (16% versus 27% respectively). Focusing on the most severe class, the impact of the severity of functional limitation significantly changes in EOSS (60% vs 100%), while it remains unchanged in EOSS-CRF (94% vs 91%). This indicates that, belonging to a severe class after BS largely depends on functional impairment, while the impact of the latter when assessed by CRF remains almost unchanged when compared to pre-BS, thus presenting itself as more reliable.

As obesity classification grading system, EOSS-CRF is novel, so its principal limitation is due to the absence of specific and long-term studies correlating it with hard endpoints, like already done for BMI and EOSS. EOSS-CRF is a new proposal with small but significant modifications, which has to be evaluated by specific trials (9, 12). Indeed, no studies have yet assessed the predictive value of the EOSS-CRF classification and a long-term follow-up project will be requested. Moreover, this study came across some limitations of the EOSS. First, psychiatric parameters are gained based on self-reported patient history data only, even though considering the Diagnostic and Statistical Manual of Mental Disorders classification. Secondly, our study has no patients grouped in EOSS/EOSS-CRF class 4, due to the small sample size, as shown in literature (48). A possible small bias of this study may consist in the inclusion of both treadmill and bicycle ergometer testing to perform CPET, with the risk of underestimation of VO_2_peak by the latter (27). Nevertheless, this was clinically conditioned and may indeed reflect functional impairment in case of walking difficulties. Moreover, one of the major strengths of our study is that all patients performed a maximal CPET, with objectively measured CRF data.

In conclusion, when combining all the evaluations performed, the multi-dimensional improvements obtained after BS in patients with severe obesity are adequately summarized by EOSS and EOSS-CRF. These classifications allow a more holistic assessment of obesity as a chronic disease both before and after bariatric surgery. Down-staging of obesity is possible with effective clinical management. We propose the inclusion of this new EOSS-CRF for representing the outcomes of obesity management in future intervention trials. EOSS-CRF grading, indeed, seems to better reflect the known amelioration that BS has demonstrated on all aspect of functional impairment and health indices. Finally, considering the functional domain, in EOSS and EOSS-CRF classifications it has a different impact in determining class assignment. Focusing on the severe included class, before and after BS this impact significantly changes in EOSS, while it remains almost unchanged in EOSS-CRF, thus presenting itself as more reliable.

## Data availability statement

The raw data supporting the conclusions of this article will be made available by the authors, without undue reservation.

## Ethics statement

The studies involving human participants were reviewed and approved by Padua Ethical Committee for Clinical Research. The patients/participants provided their written informed consent to participate in this study.

## Author contributions

All authors participated in the preparation of the manuscript and approved this submission. All authors contributed to the article and approved the submitted version.
